# Mediation effect of gut microbiota on the relationship between physical activity and carotid plaque

**DOI:** 10.3389/fmicb.2024.1432008

**Published:** 2024-07-11

**Authors:** Wenbin Ouyang, Bei Tang, Yongmei He, Hao Wu, Pingting Yang, Lu Yin, Xiaohui Li, Ying Li, Xin Huang

**Affiliations:** ^1^Department of Epidemiology, Hunan Normal University School of Medicine, Changsha, China; ^2^Department of Health Management, Aerospace Center Hospital, Beijing, China; ^3^Department of Health Management, The Third Xiangya Hospital, Central South University, Changsha, China; ^4^Medical Research & Biometrics Center, National Center for Cardiovascular Diseases, Chinese Academy of Medical Sciences and Peking Union Medical College, Beijing, China; ^5^Department of Pharmacology, Xiangya School of Pharmaceutical Science, Central South University, Changsha, Hunan, China

**Keywords:** physical activity, gut microbiota, carotid plaque, mediation, phylogenetic tree

## Abstract

**Background:**

Physical activity has been shown to have an effect on Carotid plaque (CP) which is a predictor of Cardiovascular disease (CVD). Studies have shown that physical activity can alter the composition of gut microbiota, whether its influence on CP was mediated by gut microbiota has yet to be proved.

**Methods:**

We conducted a case–control study involving 30 CP patients and 31 controls. Logistic regression was used to analyze the association between CP and physical activity. LefSe was used to explore the association between gut microbiota and physical activity as well as CP, and PhyloMed was used to examine the mediating effect of gut microbiota in the association between physical activity and CP.

**Results:**

After adjusting for potential confounders, adequate physical activity showed a significant association with a decreased risk of CP (OR_adj_: 0.25, 95%CI: 0.06, 0.97). CP was associated with enrichment in the order *Bacteroidales* within the phylum *Bacteroidetes* and the predominant microbiota in individuals without plaque was the order *Clostridiales* (LDA scores >3). Individuals with adequate physical activity had a higher abundance of the order *Clostridiales*, while the order *Bacteroidetes* was enriched in individuals with inadequate physical activity (LDA scores >3). The PhyloMed revealed a significant mediation effect of gut microbiota in the association between physical activity and CP (*p* = 0.03).

**Conclusion:**

Adequate physical activity was significantly associated with a decreased risk of CP, and this association was mediated by an increase in the abundance of gut microbiota in the order *Clostridiales*.

## Introduction

Cardiovascular disease (CVD) is a major disease burden globally, and by 2030 it is expected that approximately 26 million people will die annually from CVD (Roth et al., 2017; Song et al., 2018; Timmis et al., 2018; Song et al., 2020). The Chinese population is currently comprised of roughly 290 million individuals with CVD, which is the leading cause of death (Bei et al., 2019). Atherosclerosis is often regarded as an early stage of cardiovascular disease, with carotid atherosclerosis being a major manifestation (Wang et al., 2018; Poznyak et al., 2020; Chen et al., 2021). Carotid plaque (CP), most commonly occurring at the carotid bifurcation, is a marker for carotid atherosclerosis and a risk predictor of future atherosclerotic cardiovascular disease (Wang et al., 2018; Campaniello et al., 2022; Chen et al., 2022; Meng et al., 2023). Therefore, it is of great importance to prevent and reduce CP and understand its mechanisms to reduce the burden of CVD.

Researchers have observed that in the general population, the elderly, and those at high risk of cardiovascular disease, increased levels of physical activity are significantly and negatively correlated with the risk of CP. The underlying mechanisms may involve antithrombotic/fibrinolytic effects to reduce blood pressure or increased nitric oxide bioavailability to improve endothelial function, all of which are changes that can affect carotid artery structure and function (Chen et al., 2022). Meanwhile, a retrospective cohort study of Mexican Americans who collected metabolic equivalents found that moderate levels of physical activity were significantly negatively associated with plaque formation (Walker et al., 2019). This protective effect may stem from the ability of prolonged physical activity to reduce oxidative stress and inflammatory responses in individuals with existing atheromatous plaque, thereby slowing its progression toward cardiovascular events (Mury et al., 2018, 2019).

Recent studies have also indicated that physical activity may influence gut microbiota characteristics. For example, the gut microbiota had an entirely different compositional profile between athletes and non-athletes; increased physical activities in the general population may affect the gut microbial composition (Cronin et al., 2017; Allen et al., 2018; Aragón-Vela et al., 2021; Bonomini-Gnutzmann et al., 2022; Campaniello et al., 2022; Dziewiecka et al., 2022; Wegierska et al., 2022; Meng et al., 2023). On the other hand, the altered gut microbes diversity, distribution, and metabolites could contribute both directly and indirectly to the development of underlying cardiovascular risk factors (Karlsson et al., 2012; Hasan et al., 2019; Kiouptsi et al., 2019; Kurilshikov et al., 2019; Tang et al., 2019; Gurung et al., 2020; Szabo et al., 2021; Zhu et al., 2021) and thus play a significant role in the development of CVD. CP as an effective predictor of future atherosclerotic cardiovascular disease shares many common risk factors and biological mechanisms with CVD (Ma and Li, 2018; Shan et al., 2018; Franco-Gutiérrez et al., 2019; Al Samarraie et al., 2023). However, the role of gut microbes in the association between physical activity and CP could not be directly answered through concurrent studies. We, therefore, conducted a case–control study to examine whether the altered gut microbiota composition could be one of the mechanisms by which physical activity reduces CP risk.

## Methods

### Study the population

From Jan 2019 to Dec 2019, 30 patients diagnosed with CP and 31 controls were enrolled in the Health Management Center of Third Xiangya Hospital of Central South University. The enrollment criteria for cases were: (1) diagnosed with CP; (2) absence of coronary heart disease, cerebrovascular disease, peripheral artery disease, or aortic disease. For controls, the enrollment criteria were: (1) with negative carotid artery test results; (2) absence of coronary heart disease, cerebrovascular disease, peripheral artery disease, or aortic disease. A case or control participant was excluded if (1) had not been assessed for carotid artery; (2) had consumed anticoagulants or other medications to treat coronary heart disease cerebrovascular disease, peripheral artery disease, or aortic disease; (3) had consumed laxatives, probiotics, anti-diarrheal medications, immune-suppressants, anticancer agents or antibiotics within the preceding 3 months; (4) had not attended the gut microbiota examination. All participants received physical and laboratory examinations and were asked to complete self-report questionnaires detailing their socioeconomic status, health-related habits, daily physical activity, and medical history. Blood samples were collected to measure fasting serum glucose (FSG), total cholesterol (TC), triglyceride (TG), low-density lipoprotein cholesterol (LDL-C), and high-density lipoprotein cholesterol (HDL-C) levels. The study received approval from the Ethics Committee of the Third Xiangya Hospital of Central South University. All participants signed an informed consent form.

### International Physical Activity Questionnaire (IPAQ-S-C)

The ten-question IPAQ-S-C, adapted according to the short version of the International Physical Activity Questionnaire (Hagströmer et al., 2008), was used to assess habitual physical activity during the past seven days. The IPAQ-S-C evaluates four items, including vigorous intensive, moderate intensive, walking, and sedentary activities. Using 1.0 metabolic equivalent (MET) for sedentary activity, 3.3 METs for walking, 4.0 METs for moderate physical activity excluding walking, and 8.0 METs for vigorous activity. The corresponding physical activity was sorted into time (h/week) or energy (MET-h/week) formats. According to individual physical activity, participants were categorized into “low,” “medium” and “high” levels of physical activity. Low levels of physical activity were defined in our study as inadequate, while medium or high levels of physical activity were defined as adequate.

### Carotid plaque assessment

The carotid artery was assessed using the Siemens Acuson SequoiaTM 512 Ultrasound System (Mountain View, CA, USA) with a linear matrix array transducer operating at 12 MHz. In a supine position, participants were examined by trained and experienced ultrasound doctors. A carotid artery intima-media thickness (IMT) was determined by measuring the distance between the leading edge of a lumen-intima echo and the leading edge of a media-adventitia echo, as measured on transverse and sagittal projections of the common carotid artery (CCA) and bifurcation carotid artery (BCA). Carotid IMT exceeding 1.5 mm or focal thickening greater than 50% of the surrounding wall thickness are considered cases of CP.

### Genomic DNA extraction, library construction, and sequencing

Participants were asked to collect fecal samples on the same day they had a physical examination. These samples were stored at −80°C and then DNA extracted within a week. Total genomic DNA was extracted from each sample using the MagPure Stool DNA KF kit B (Magen, Guangzhou, China) according to the product instructions, and measured using a Quantum Bit Fluorometer (Qubit 2.0, Invitrogen, CA, USA) and the Quantum Bit® dsDNA BR Assay Kit (Invitrogen). DNA and DNA quality were determined using gel electrophoresis (1% agarose gel). A negative control for DNA extraction and quantification consisting only of an elution buffer was used to check for potential contamination. Polymerase chain reaction for amplification of the hypervariable region V4 of the bacterial 16S rRNA gene was performed using the Taq PCR Master Mix (Thermo Fisher Scientific, USA) kit according to the product instructions. After amplification was completed, amplicons were purified using the Nucleic Acid Purification Kit (Beckman Coulter, California, USA) according to the product instructions. The library quality was determined using a High Sensitivity DNA chip on the 2100 Bioanalyzer (Agilent Technologies, California, USA). Sequencing was conducted on the HiSeq 2500 platform (BGI, Shenzhen, China) utilizing the standard Illumina sequencing workflow to generate two paired-end reads of 250 bp. Sequencing results are distinguished for each sample by the barcode tags added before sequencing. Sequences that meet quality control requirements will be spliced according to overlap. Finally, optimized sequences are obtained by filtering out non-compliant sequences from spliced sequences and then classified into operational taxonomic units (OTUs). Following the annotation of species information based on the OTU data, the dataset was homogenized according to taxonomic classification at various levels. Specifically, the classification included phylum, order, family, genus, and species. Subsequently, the relative species percentage was calculated independently for each sample at these six taxonomic levels.

### Statistical analysis

The distributions of demographics, physical activity, and clinical characteristics were compared between cases and controls using t-tests and chi-square tests for continuous variables and categorical variables, respectively. Statistical significance was assessed at the 5% level (two-tail test). Logistic regression was used to analyze the association between physical activity and CP. The clustering of OTUs resulted in 633 OTUs, with 503 OTUs in the CP group, 573 OTUs in the control group, and 443 OTUs in both groups. A total of 12 phyla, 18 classes, 30 orders, 39 families, 51 genera, and 90 species have been identified following OTUs annotation. Association studies were conducted at the species level. The differences in gut microbial species diversity and abundance between case and control groups were examined at the species level using α-diversity indices (Shannon, Chao-1, and Simpson) and β-diversity-weighted UniFrac distances. Linear discriminant analysis Effect Size (LefSe) was used to explore the association between gut microbial species and physical activity as well as CP. Linear Discriminant Analysis (LDA) was used to estimate the LDA score for each species, where LDA scores greater than 2.0 were used as significantly enriched species. An integrated phylogenetic analysis was conducted to ascertain the evolutionary order of gut microbial species that exhibited significant differences in abundance between the two groups. In the present study, we excluded species present in less than 20% of the population or with an average relative abundance of less than 0.001%. We used the PhyloMed model to investigate whether certain gut bacteria mediate the protective effect of physical activity on CP. In mediation analyses, we adjusted for multiple comparisons using a false discovery rate (FDR) of 10% (< 0.10) for the average causal mediated effect tests. R 4.2.3 was used to conduct the statistical study.

## Results

In total, 30 subjects with CP and 31 controls were enrolled and analyzed. No statistically significant differences were observed in the distribution of age, sex, year of education, marriage status, smoking habits, alcohol usage, hypertension, diabetes, dyslipidemia, systolic blood pressure (SBP), diastolic blood pressure (DBP), or body mass index (BMI), between CP cases and controls (*p* > 0.05, Table 1). However, the CP group had lower levels of HDL-C and higher levels of TC, TG, LDL-C, and FSG (*p* < 0.05). Using the number of OTUs as the coordinate and the number of samples taken as the abscissa, we constructed a rarefaction curve. There was a sharp rise in the rarefaction curve at the beginning and then gradually flattened out, indicating that the sequencing data volume and sequencing depth of the samples were reasonable (Supplementary Figure S1).

**Table 1:** Characteristics of enrolled participants (*N* = 61).

Characteristics		Control (*n* = 31)	Carotid plaque (*n* = 30)	*P*
Age (years) (mean, SD)*		57.52 ± 7.59	60.17 ± 8.63	0.21
Sex (*n*, %)^†^	Males	21 (67.74)	22 (73.33)	0.63
	Females	10 (32.26)	8 (26.67)	
Year of Education (*n*, %)^†^	≤ 12 years	11 (35.49)	15 (50.00)	0.25
	> 12 years	20 (64.51)	15 (50.00)	
Marriage (*n*, %)^†^	Unmarried	1 (3.23)	2 (6.67)	0.53
	Married	30 (96.77)	28 (93.33)	
Smoke (*n*, %)^†^	No	23 (74.19)	17 (56.67)	0.15
	Yes	8 (25.81)	13 (43.33)	
Alcohol (*n*, %)^†^	No	22 (70.97)	16 (53.33)	0.16
	Yes	9 (29.03)	14 (46.67)	
Hypertension (*n*, %)^†^	No	24 (77.42)	21 (70.00)	0.51
	Yes	7 (22.58)	9 (30.00)	
Diabetes (*n*, %)^†^	No	26 (83.87)	20 (66.67)	0.12
	Yes	5 (16.13)	10 (33.33)	
Dyslipidemia (*n*, %)^†^	No	17 (54.84)	10 (33.33)	0.09
	Yes	14 (45.16)	20 (66.67)	
TC (mmol/L) (mean, SD)*		4.94 ± 1.11	5.85 ± 0.97	< 0.01
TG (mmol/L) (mean, SD)*		1.53 ± 0.85	1.96 ± 0.60	0.03
LDL⁃C (mmol/L) (mean, SD)*		2.69 ± 0.81	3.20 ± 0.80	0.02
HDL⁃C (mmol/L) (mean, SD)*		1.45 ± 0.31	1.28 ± 0.26	0.03
FSG (mmol/L) (mean, SD)*		5.50 ± 0.69	6.26 ± 1.68	0.02
SBP (mmHg) (mean, SD)*		126.83 ± 18.06	129.77 ± 14.30	0.49
DBP (mmHg) (mean, SD)*		79.57 ± 12.62	79.00 ± 9.54	0.84
BMI (kg/m^2^) (mean, SD)*		24.62 ± 3.35	25.15 ± 2.62	0.50

*Compared by t-test; ^†^compared by chi-square test.

### Carotid plaque and physical activity

Only 40.00% of CP cases (12/30) were identified as having adequate physical activity, the control group showed a higher percentage of 61.29% (19/31). This difference, however, was statistically insignificant between the two groups (*p* > 0.05, OR: 0.42, 95%CI: 0.15, 1.17). When the variables of age, sex, education, marital status, smoking habits, alcohol usage, BMI, and medical history of hypertension, diabetes, and dyslipidemia were adjusted for confounding effects, adequate physical activity was significantly associated with a decreased risk of CP (OR_adj_: 0.25, 95%CI: 0.06, 0.97) (Table 2).

**Table 2:** Association between physical activity and carotid plaque.

Physical activity	Control (*n* = 31)	Carotid plaque (*n* = 30)	OR (95%CI)	*OR _adj_ (95%CI)
Inadequate (*n*, %)	12 (38.71)	18 (60.00)	Ref.	Ref.
Adequate (*n*, %)	19 (61.29)	12 (40.00)	0.42 (0.15, 1.17)	0.25 (0.06, 0.97)

*Adjustment variables include age, sex, education, marriage, smoke, alcohol, hypertension, diabetes, dyslipidemia, and BMI.

### Gut microbiota and carotid plaque

**Figure 1:**
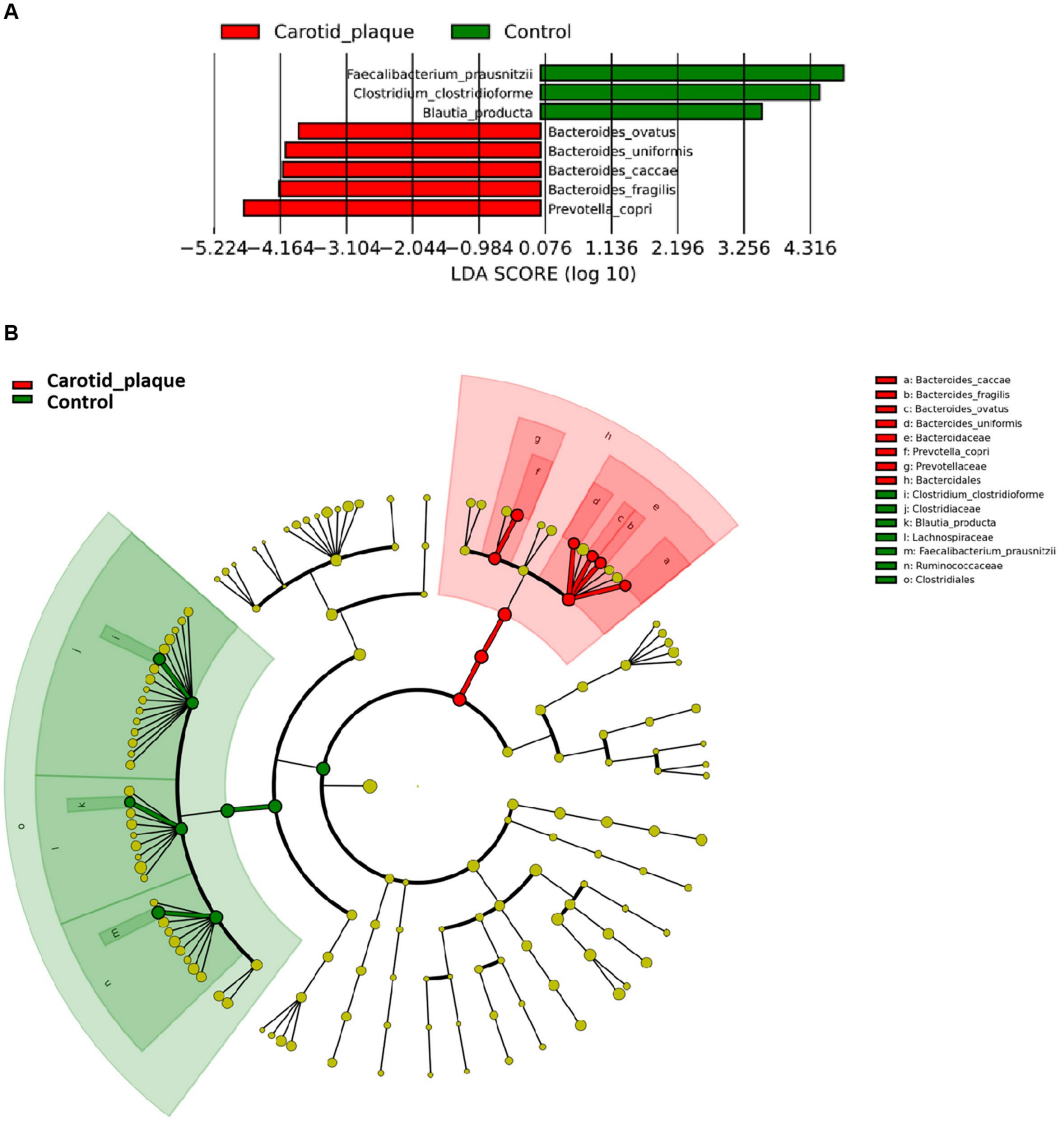
Different abundance species according to carotid plaque status. **(A)** Presents the distribution of LDA (Linear Discriminant Analysis) values for different species, with colors representing corresponding groups. **(B)** Illustrates the phylogenetic tree of the differentially abundant species, with concentric circles radiating from the center representing taxonomic levels ranging from phylum to genus (or species). Each small circle at different taxonomic levels represents a specific classification, and the diameter of the circle is proportional to its relative abundance. The coloring scheme follows certain principles: non-significant species are uniformly colored in yellow, differentially abundant species are colored based on their respective groups, and red nodes represent microbial taxa that play a significant role within the red group, while green nodes represent microbial taxa that play a significant role within the green group.

The Shannon index, Chao-1 index, and Simpson index did not show significant differences between the CP and the control group, meaning that there was no statistically significant difference in α diversity (Supplementary Figure S2A). Additionally, the comparison of β diversity showed similar results (R^2^ < 0.1) (Supplementary Figure S2B). LefSe analysis revealed that CP was associated with enrichment in *Bacteroides ovatus*, *Bacteroides uniformis*, *Bacteroides caccae*, *Bacteroides fragilis*, and *Prevotella copri* (LDA scores >3, Figure 1A) which belong to the order *Bacteroidales* within the phylum *Bacteroidetes* (Figure 1B). Meanwhile, CP was associated with a decrease in *Faecalibacterium prausnitzii*, *Clostridium clostridioforme*, and *Blautia producta*. These species all belong to the same order *Clostridiales* within the phylum *Firmicutes*.

Concurrently, we also examined the associations between these CP-related species and traditional CVD risk factors. The results show that a positive association was observed between *B. caccae* and TC (*r* = 0.26, *p* = 0.04), and a negative association between *C. clostridioforme* and TC (*r* = −0.37, *p* < 0.01). Additionally, notable negative associations were also discerned between *B. producta* (*r* = −0.29, *p* = 0.02), *C. clostridioforme* (*r* = −0.36, *p* < 0.01) and TG (Supplementary Figure S3).

### Gut microbiota and physical activity

**Figure 2:**
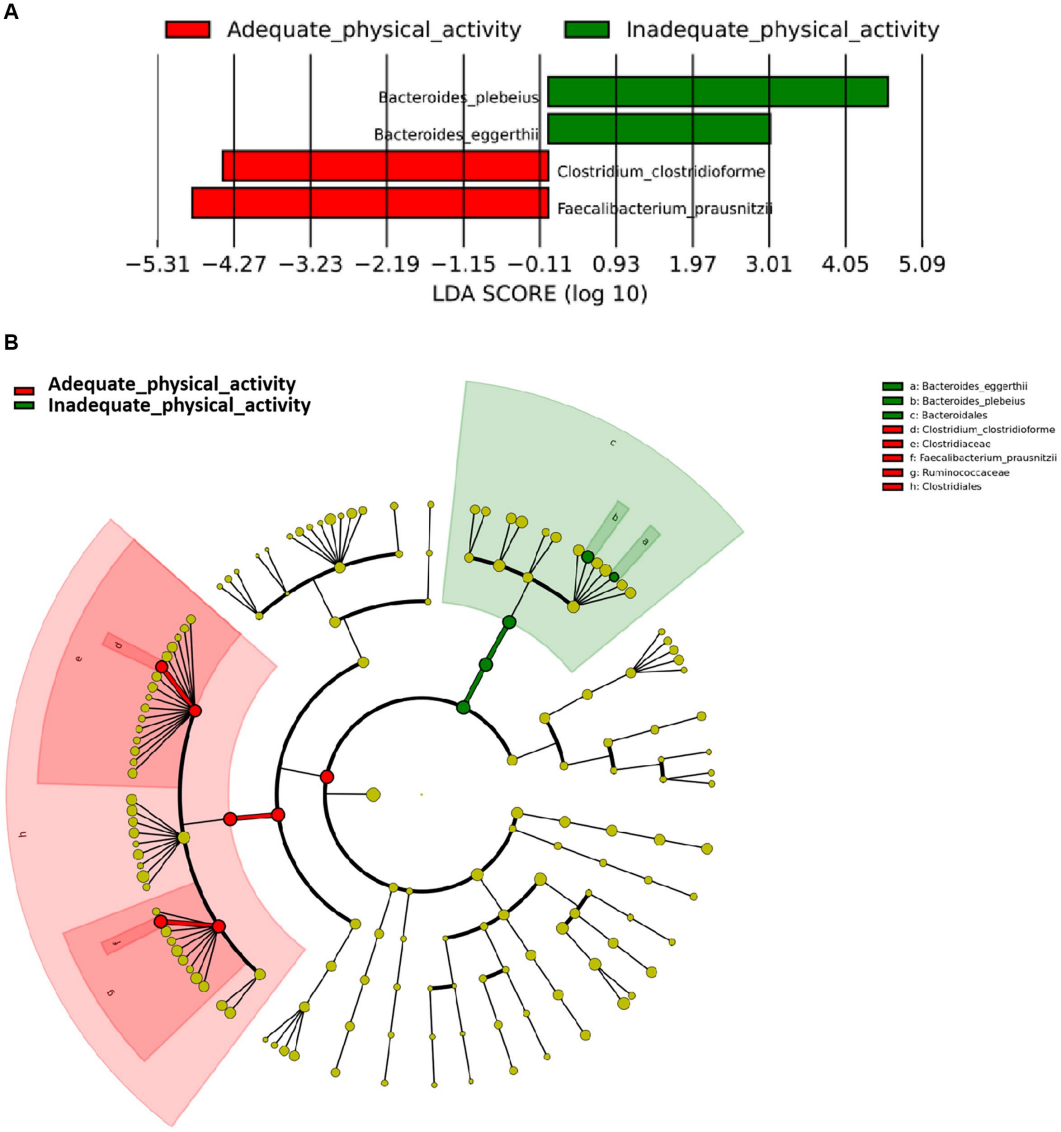
Differentially abundant species according to physical activity status. **(A)** Presents the distribution of LDA (Linear Discriminant Analysis) values for different species, with colors representing corresponding groups. **(B)** Illustrates the phylogenetic tree of the differentially abundant species, with concentric circles radiating from the center representing taxonomic levels ranging from phylum to genus (or species). Each small circle at different taxonomic levels represents a specific classification, and the diameter of the circle is proportional to its relative abundance. The coloring scheme follows certain principles: non-significant species are uniformly colored in yellow, differentially abundant species are colored based on their respective groups, and red nodes represent microbial taxa that play a significant role within the red group, while green nodes represent microbial taxa that play a significant role within the green group.

It was found that individuals with adequate physical activity were enriched in *Faecalibacterium prausnitzii* and *Clostridium clostridioforme* (LDA scores >3, Figure 2A) which both belonged to the same order *Clostridiales* within the phylum *Firmicutes* (Figure 2B). On the other hand, individuals with inadequate physical activity were enriched in *Bacteroides eggerthii* and *Bacteroides plebeius* which belonged to the order *Bacteroidales* within the phylum *Bacteroidetes*.

### Mediation role of gut microbiota in the association between physical activity and carotid plaque

**Figure 3:**
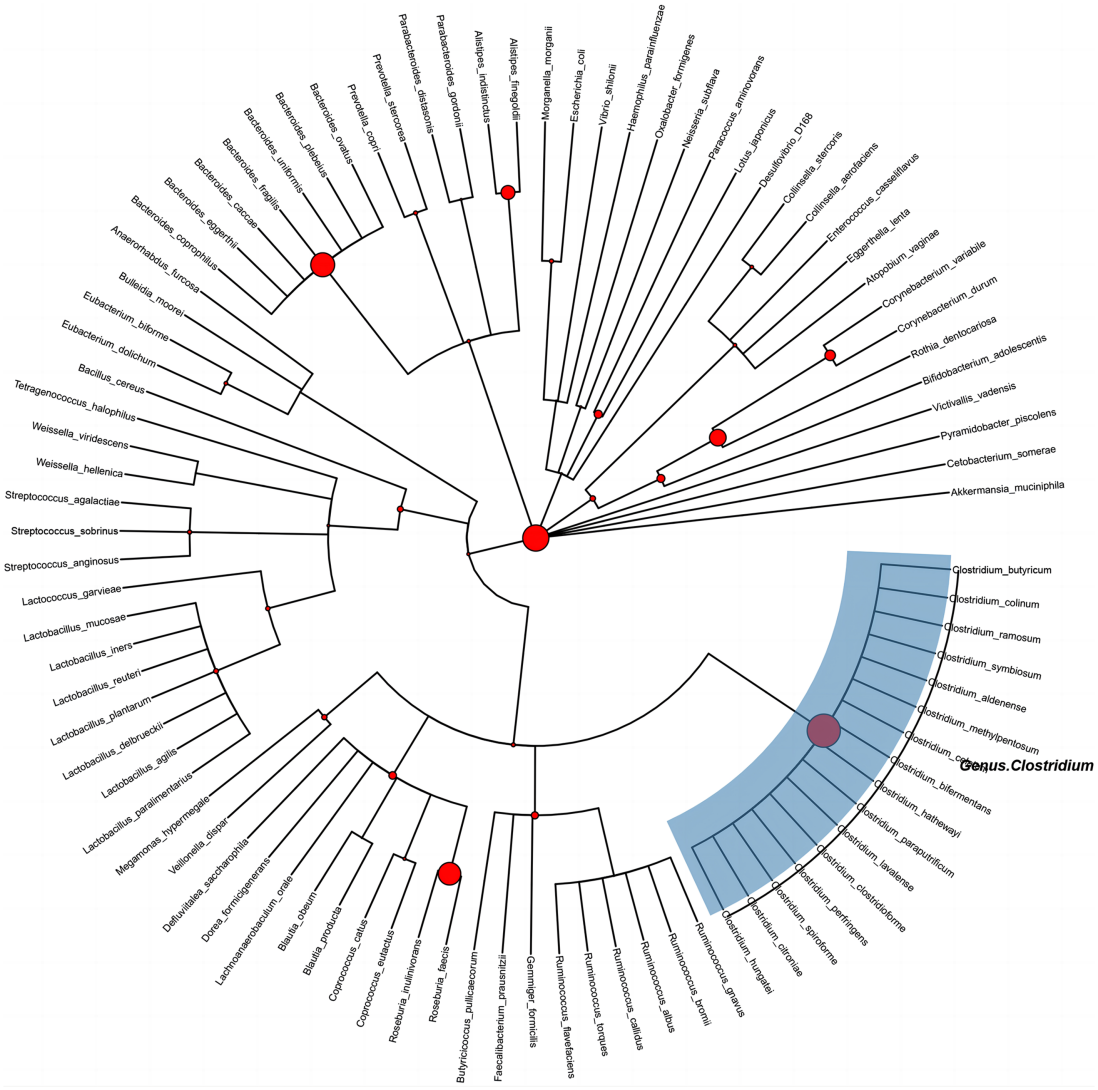
Phylogenetic tree of gut microbes between physical activity and carotid plaque effects. Circle size at each internal node is proportional to −log 10 (PhyloMed local mediation test *p*-value). The identified intermediary internal nodes have the largest red circles, and subtrees under nodes with two OTU descendants are highlighted with blue rectangles.

After the adjustment of the potential confounding factors, the PhyloMed global mediation test revealed a significant mediation effect of gut microbiota in the association between physical activity and CP (*p* = 0.03) (Figure 3). The identified internal nodes and child nodes were leaf nodes comprising multiple OTUs, and they were all classified under the genus *Clostridium*. This finding suggested that adequate physical activity may reduce the likelihood of CP development by increasing the abundance of *Clostridium*.

## Discussion

In our study, we investigated the association between physical activity and gut microbiota and examined the role that gut microbiota played in mediating the association between physical activity and CP. It was found that physical activity significantly influenced the composition of the gut microbiota. The abundance of *F. prausnitzii* and *C. clostridioforme* in individuals increased dramatically in those who were physically adequate, which is consistent with previous studies. Bressa et al. (2017) conducted a study on women who performed physical activity levels that were up to standard and found that *F. prausnitzii* abundance was positively correlated with physical activity levels. O’Donovan et al. (2020) showed that high-level athletes with an intestinal relative abundance of *F. prausnitzii* would be higher compared to the general population, while the abundance of *Faecalibacterium* and species *Clostridium* has also been shown to be increased in bodybuilders (Jang et al., 2019).

Our results also indicate that individuals who achieve a standard level of physical activity have a significantly lower risk of developing CP. Previous studies have demonstrated that physical activity can reduce the level of inflammatory response and oxidative stress in the body, thus favoring cardiovascular health and slowing down the progression of CP through mechanisms such as lowering LDL concentration, improving endothelial function, reducing the expression of adhesion molecules, and decreasing macrophage/leukocyte infiltratio (Mury et al., 2018). In our study, we also explored the possible mechanisms of the protective effect of physical activity on CP. We found that both *F. prausnitzii* and *C. clostridioforme*, which were enriched in individuals who had attained physical activity, could produce short-chain fatty acids (SCFA), and that both colonies belonged to the order *Clostridiales* (Choi et al., 2023). SCFA has demonstrated an important role in reducing systemic inflammation and improving intestinal barrier integrity. The mechanisms may include stimulation of G protein-coupled receptors, inhibition of NF-kB activation, and upregulation of tight junction proteins (Dalile et al., 2019). By inhibiting systemic chronic inflammation, SCFA significantly reduces lipid accumulation, foam cell formation, smooth muscle cell proliferation, endothelial dysfunction, and collagen degradation in the arterial wall, thereby inhibiting CP formation and disrupting more vulnerable plaques (de Vos et al., 2022). These imply that order *Clostridiales* may play a role in protecting against CP through the production of SCFA and thus physical activity.

Previous studies have investigated the effect of Clostridia-associated microbiota on CP. Menni et al. (2018) found a negative correlation between pulse wave conduction velocity in females and their clostridial microbiota abundance, which therefore indicates that lower clostridial microbiota abundance may contribute to CP formation. A study conducted on women with HIV or at high risk of HIV also showed that the four species negatively associated with CP belong to the order *Clostridiales* within the phylum *Firmicutes* (Wang et al., 2023). As well, Novakovic et al. (2020) noted the association of plaque formation with direct infection by species *Clostridium*. All of the studies have implied a link between CP and gut microbiota, especially the order *Clostridiales*, which is consistent with our findings.

Besides the order *Clostridiales*, we also found that the microbiota of the phylum *Bacteroidetes* is also involved in the role of physical activity on CP. The results of our study are consistent with previous studies in which Mokhtarzade et al. (2022) found a significant negative correlation between the level of physical activity and the abundance of phylum *Bacteroidetes* in patients with multiple sclerosi, and Jonsson and Bäckhed (2017) showed that the abundance of phylum *Bacteroidetes* bacteria would be higher in patients with CP. These studies indicate that the microbiota of the phylum *Bacteroidetes* may also plays a role in the pathway of action of physical activity on CP.

Combined with the results of the mediator analysis, we found that among many gut microbiota, species *Clostridium* may play an important mediating role in the association between physical activity and CP. We hereby consider that individuals may inhibit CP formation after physical activity by altering the composition of the gut microbiota and increasing the abundance of SCFA-producing gut microbiota, particularly the order *Clostridiales*, to reduce systemic inflammation.

Our study has several strengths. By examining the mechanisms by which physical activity reduces CP, we added direct proof to the field. The findings of this study will have substantial clinical implications for the prevention of plaque formation. It is possible, for example, to reduce plaque formation similarly to increasing physical activity by supplementing relevant gut microbiota in insufficiently physically active individuals with limited mobility. Furthermore, the data contained information on health behaviors and sociodemographic characteristics allowing adjustment for these potential confounding factors. Limitations should also be considered when interpreting the results. Although we have considered medical and demographic information, the dataset contains no information on diet consumption. As a result, residual confounding in our study results may still exist. Our study was a case–control study and the data on exposure and outcome in our study were collected at the same period thus the causal link between physical activity and the carotid plaque had not been demonstrated in the current study. Moreover, our study was conducted among Chinese with a relatively small sample size, so the replication of our findings in an independent larger population is needed. Lacking data on circulating short-chain fatty acids and inflammatory cytokine levels also limits further insight into the detailed mechanisms of gut microbial action. The multi-omics approach combining macro-transcriptomics, metabolomics, and proteomics is needed to further elucidate the complex mechanisms of crosstalk between physical activity, the gut microbiome, and cardiovascular health outcomes.

## Conclusion

This study found that adequate physical activity may reduce the likelihood of CP development by increasing the order of *Clostridiales* abundance. The findings may help us better understand the link between physical activity, gut microbiota, and vascular health, while also providing hints for developing microbiome-targeted therapies to slow the progression of atherosclerosis in the future.

## Data availability statement

The original contributions presented in the study are included in the article/Supplementary material, further inquiries can be directed to the corresponding authors.

## Ethics statement

The studies involving humans were approved by Ethics Committee of the Third Xiangya Hospital of Central South University. The studies were conducted in accordance with the local legislation and institutional requirements. The participants provided their written informed consent to participate in this study. Written informed consent was obtained from the individual(s) for the publication of any potentially identifiable images or data included in this article.

## Author contributions

WO: Writing – original draft, Formal analysis. BT: Writing – original draft. YH: Writing – review & editing, Supervision, Investigation. HW: Writing – review & editing. PY: Writing – review & editing, Investigation. LY: Writing – review & editing, Formal analysis. XL: Writing – review & editing. YL: Writing – review & editing, Supervision, Methodology, Funding acquisition. XH: Writing – review & editing, Supervision, Methodology, Funding acquisition.
