# Supplementary figures and images for: Mediation effect of gut microbiota on the relationship between physical activity and carotid plaque
Source: Front Microbiol. 2024 Jul 11;15:1432008. doi: 10.3389/fmicb.2024.1432008 (PMC11269180; doi:10.3389/fmicb.2024.1432008)

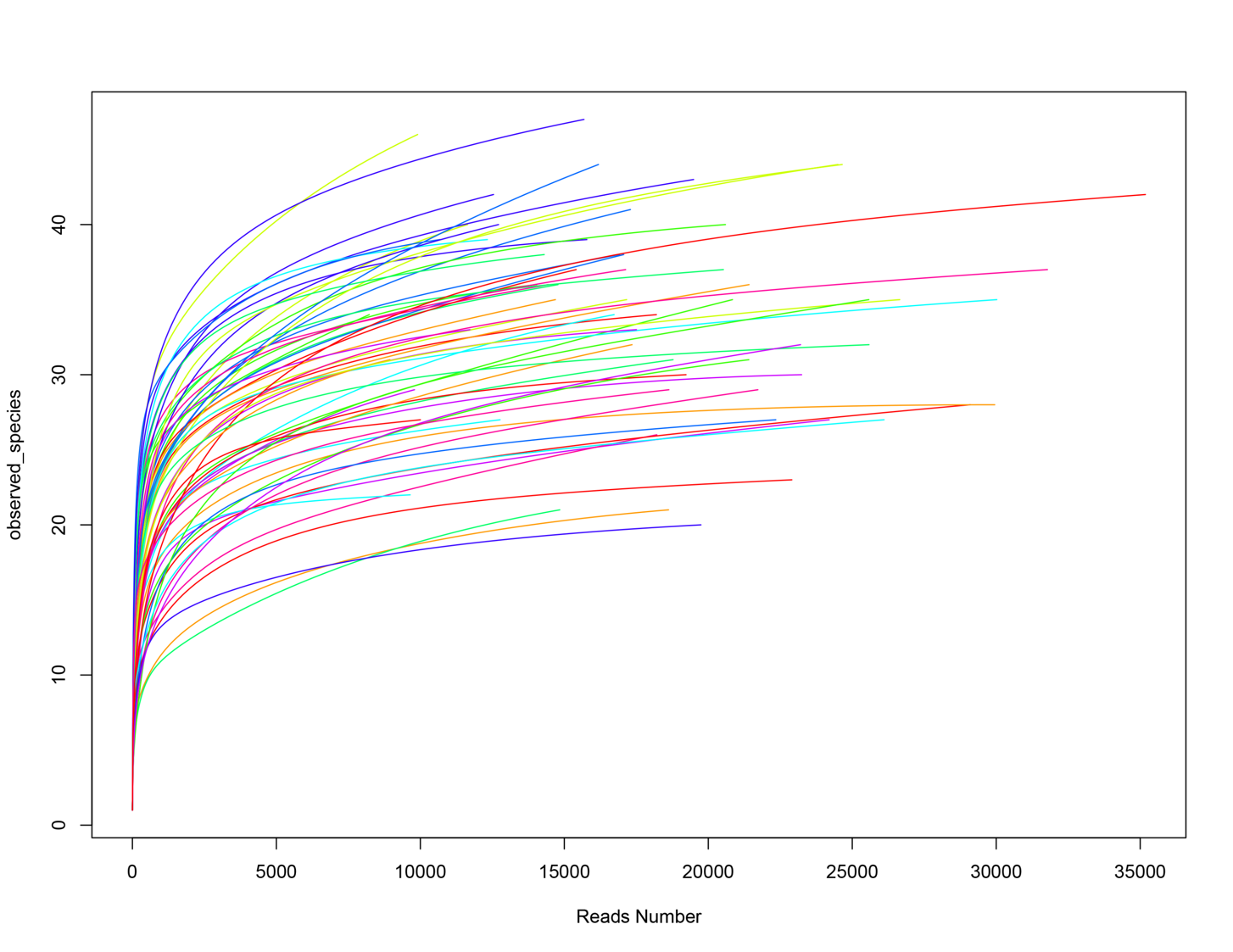

Supplement: Supplementary file 1 [file Image_1.PNG]

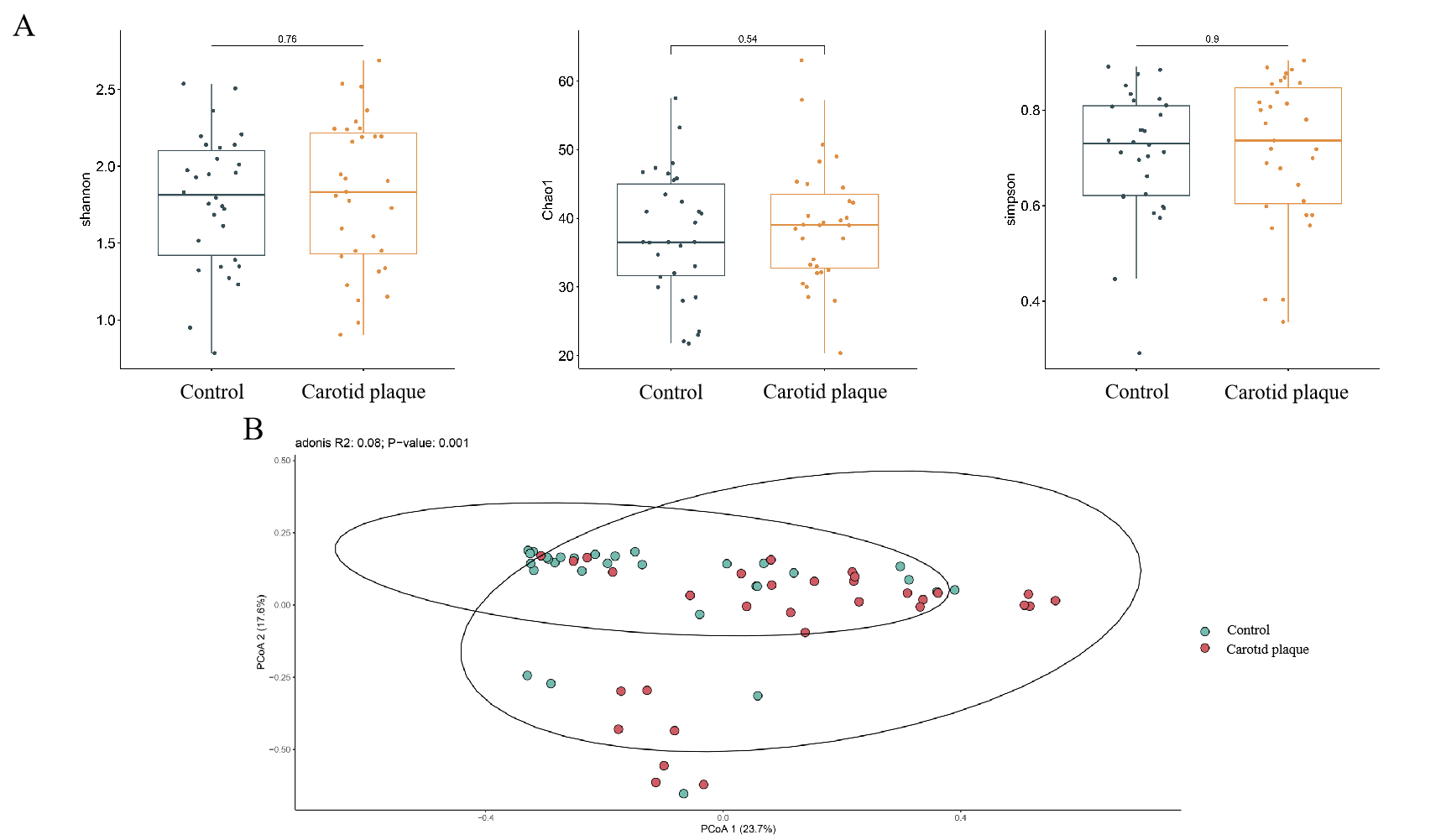

Supplement: Supplementary file 2 [file Image_2.PNG]

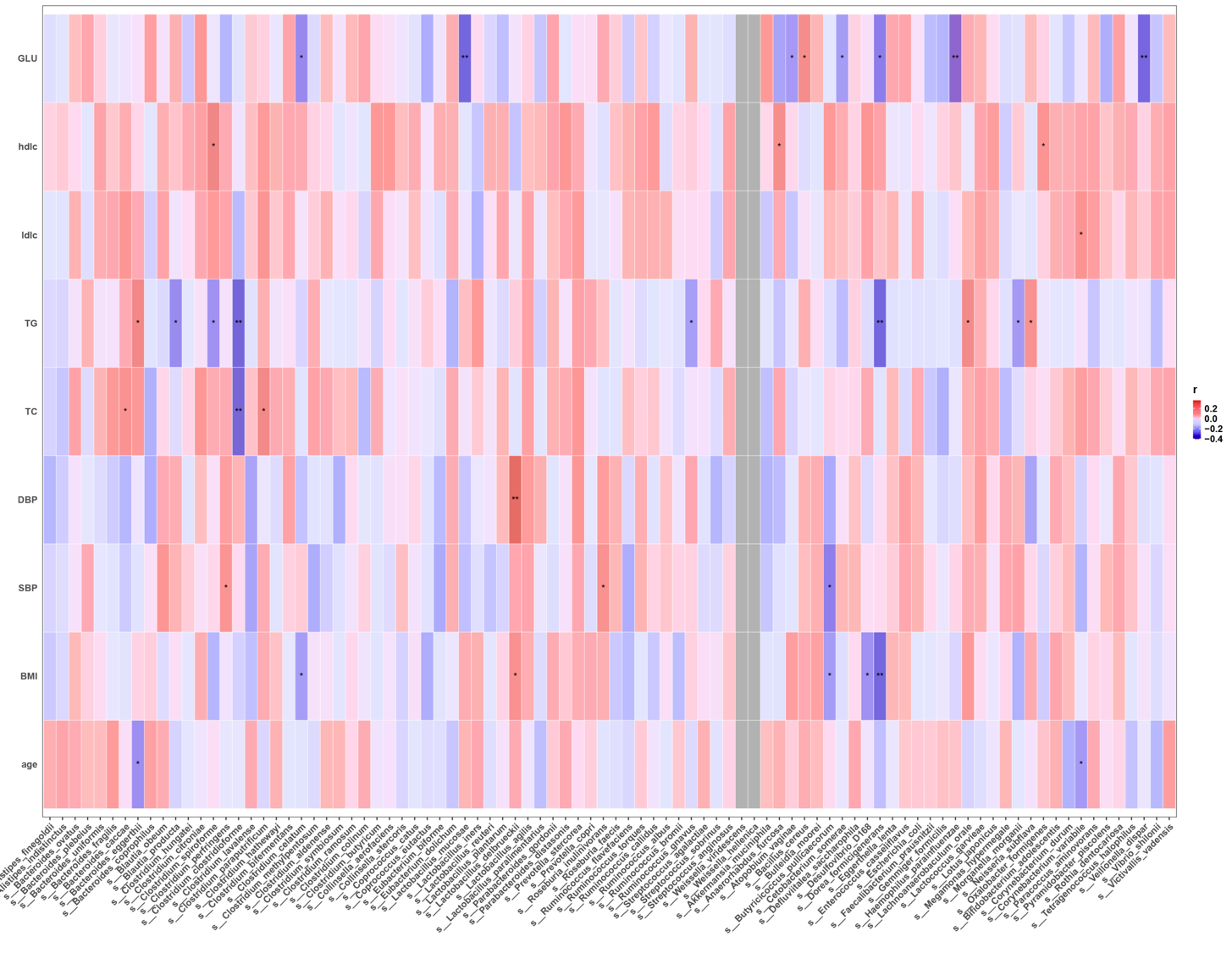

Supplement: Supplementary file 3 [file Image_3.PNG]
